# Exploring the causal relationship between gut microbiota and frailty: a two-sample mendelian randomization analysis

**DOI:** 10.3389/fmed.2024.1354037

**Published:** 2024-05-03

**Authors:** Fuduo Bo, Hong Teng, Jianwei Shi, Zhengxiang Luo, Yang Xu, Ruihan Pan, Yan Xia, Shuaishuai Zhu, Yansong Zhang, Wenbin Zhang

**Affiliations:** ^1^Department of Neurosurgery, The Affiliated Brain Hospital of Nanjing Medical University, Nanjing, China; ^2^Department of Geriatrics, The Affiliated Brain Hospital of Nanjing Medical University, Nanjing, China; ^3^Department of Neurosurgery, Xuanwu Hospital, Capital Medical University, Beijing, China; ^4^Department of Neurosurgery, The Second Affiliated Hospital of Nanjing Medical University, Nanjing, China

**Keywords:** gut microbiota, frailty, Mendelian randomization, elderly people, causality

## Abstract

**Background:**

Frailty is a complex geriatric syndrome that seriously affects the quality of life of older adults. Previous observational studies have reported a strong relationship of frailty with the gut microbiota; however, further studies are warranted to establish a causal link. Accordingly, we aimed to conduct a bidirectional Mendelian randomization study to assess the causal relationship between frailty, as measured by the frailty index, and gut microbiota composition.

**Methods:**

Instrumental variables for the frailty index (*N* = 175, 226) and 211 gut bacteria (*N* = 18,340) were obtained through a genome-wide association study. A two-sample Mendelian randomization analysis was performed to assess the causal relationship of gut microbiota with frailty. Additionally, we performed inverse Mendelian randomization analyses to examine the direction of causality. Inverse variance weighting was used as the primary method in this study, which was supplemented by horizontal pleiotropy and sensitivity analyses to increase confidence in the results.

**Results:**

*Bacteroidia* (*b* = −0.041, SE = 0.017, *p* = 0.014) and *Eubacterium ruminantium* (*b* = −0.027, SE = 0.012, *p* = 0.028) were protective against frailty amelioration. Additionally, the following five bacteria types were associated with high frailty: *Betaproteobacteria* (*b* = 0.049, SE = 0.024, *p* = 0.042), *Bifidobacterium* (*b* = 0.042, SE = 0.016, *p* = 0.013), *Clostridium innocuum* (*b* = 0.023, SE = 0.011, *p* = 0.036), *E. coprostanoligenes* (*b* = 0.054, SE = 0.018, *p* = 0.003), and *Allisonella* (*b* = 0.032, SE = 0.013, *p* = 0.012). Contrastingly, frailty affected *Butyrivibrio* in the gut microbiota (*b* = 1.225, SE = 0.570, *p* = 0.031). The results remained stable within sensitivity and validation analyses.

**Conclusion:**

Our findings strengthen the evidence of a bidirectional causal link between the gut microbiota and frailty. It is important to elucidate this relationship to optimally enhance the care of older adults and improve their quality of life.

## 1 Introduction

Frailty is a multifactorial syndrome that may be a physiological precursor and etiology of disability and is characterized by reduced physical strength, endurance, and physiological functioning. As the function of multiple physiological systems declines, frail individuals are less able to cope with stressors and are therefore more susceptible than healthy individuals to adverse outcomes, such as increased risk of falls, hospitalization, disability, and premature death (1). Compared with age alone, frailty is more reliable in predicting adverse health outcomes, including hospitalization, dependency, and premature death (2). Although the prevalence of frailty remains unclear, several studies have reported a gradual increase in its prevalence with age (3, 4). A systematic review of 61,500 community-dwelling older adults living in high-income countries reported a frailty prevalence of 10.4%. Nevertheless, there were varying reported prevalences among the included studies (4.0–59.1%) given the lack of reliable concept definitions or measurement standardizations (5). Frailty is poorly understood, often unrecognized in the clinical setting, and may initially be overlooked or misinterpreted as a change in the normal aging process. There are no specific medications that significantly improve frailty, and non-pharmacological interventions, e.g., nutrition and physical activity, remain a mainstay of frailty prevention and treatment (6). Increasing morbidity and poor prognosis have made frailty a major public health challenge worldwide, placing an enormous burden on society and families.

The pathogenesis of frailty involves pathophysiological processes in multiple systems, such as chronic inflammation and immune activation, as well as in the musculoskeletal and endocrine systems (7). Although in previous studies gut microbiota (GM) was found to be a major player in host nutrition, metabolism, immunity, and neurologic function, its imbalance has been strongly associated with a high risk of various health disorders, e.g., osteoporosis, autoimmune inflammatory diseases, and cardiovascular disease (8–10). In particular, GM-derived metabolites of short-chain fatty acids (SCFAs), tryptophan, lipopolysaccharides, and bile acids are closely associated with diseases, such as obesity, metabolic syndrome, and type 2 diabetes mellitus during pregnancy, by modulating the differentiation and function of inflammatory immune cells and pro-inflammatory cytokines, and they play a key role in maintaining intestinal and systemic homeostasis (11–13). Therefore, on the basis of the wide-ranging effects of gut flora on the entire body, it is reasonable to hypothesize that there may be a potential link to frailty. Recent studies have highlighted the influence of the GM on skeletal muscle (14); specifically, gut flora has been shown to regulate metabolic homeostasis and insulin sensitivity in skeletal muscle and to promote an inflammatory response in skeletal muscle, which in turn affects many aspects of muscle, including mass, function, and metabolic processes (14, 15). Subsequent skeletal muscle changes have been linked to loss of independence and reduced quality of life; further, they may comprise the underlying cause of frailty.

Although these previous studies have observed an association between GM and frailty, their bacterial results were inconsistent, the sample sizes were small, and the pathways and magnitude of the resulting effects remain unclear, which hampers the interpretation of whether frailty is a cause or a consequence of an imbalance in the gut flora (16). Additionally, the pathophysiology of frailty is intricate and encompasses multiple interrelated routes that remain unclear.

The limitations of traditional designs mean that current observational studies cannot ascertain whether reverse causality and confounding factors affect the findings, ultimately leading to skewed associations or conclusions (17). Additionally, it is unethical and impractical to conduct randomized controlled trials (RCTs) given the substantial need for human resources and lengthy follow-up periods (18, 19). Mendelian randomization (MR) is based on the principle that alleles are randomly assigned during gamete formation; further, it uses exposure-related genetic variation as an instrumental variable (IV) to assess whether there is a causal relationship between the exposure and outcome (20). Drawing on the inherent nature of genetic variation, MR analyses can effectively eliminate confounding factors and identify the causal determinants of particular outcomes (21). Consequently, MR has been increasingly used to infer feasible causal relationships between risk factors and disease outcomes.

Accordingly, we used a two-sample bidirectional MR approach to investigate the causal relationship between the GM and frailty indices to elucidate whether the microbiota influences frailty development at the genetic level and to identify the specific microorganisms involved. Exploring the potential link between GM and frailty as well as elucidating the underlying mechanisms of frailty may facilitate specific monitoring of at-risk populations, and in turn, alleviate frailty in the elderly population through adjustment of the structural composition of the GM.

## 2 Materials and methods

### 2.1 Study design

**Figure 1:**
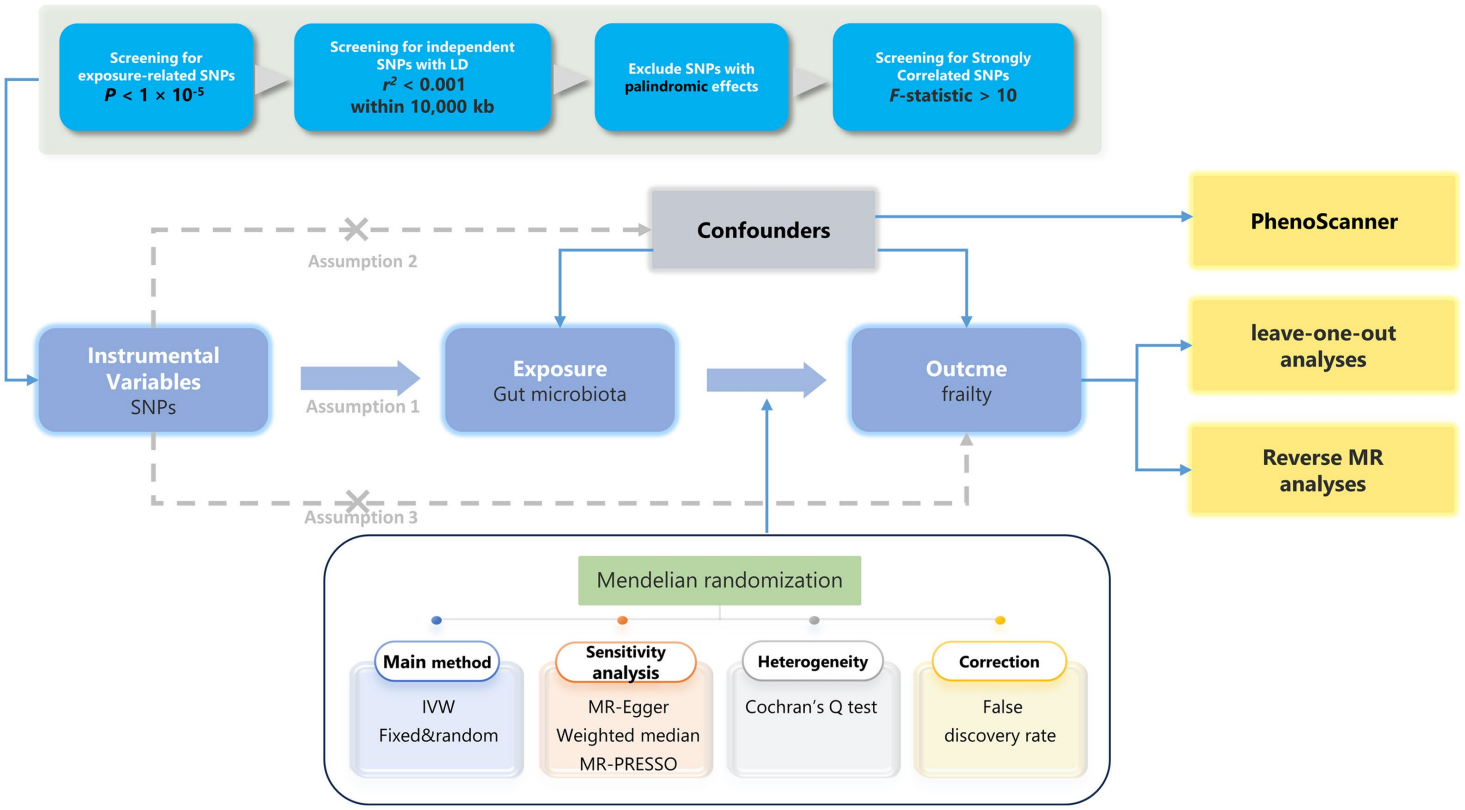
The analytical process and key assumptions of this MR study. LD, Linkage disequilibrium; MR, Mendelian randomization; SNP, single nucleotide polymorphism; IVW, inverse-variance weighted.

In this study, we performed a two-sample MR analysis using pooled genome-wide association study (GWAS) data to verify the causal relationship between the GM and frailty (the research process is shown in Figure 1). This analysis was similar to a previous RCT in that single nucleotide polymorphisms (SNPs) in the offspring were randomly assigned (confounders, such as sex and age, were excluded). Additionally, the validity of MR analyses relies on three fundamental assumptions that must be fulfilled to draw reliable conclusions: (1) genetic variation (IV) is associated with the exposure (GM) (relevance assumption); (2) genetic variation is associated with the outcome (frailty) only through exposure (exclusion restriction assumption); and (3) genetic variation is independent of all other factors affecting the outcome (independence assumption) (22).

### 2.2 GWAS data of the GM

GM-related data were obtained from the MiBioGen Consortium’s large-scale GWAS, which is currently the largest microbiome meta-analysis in the world. This large-scale study included 18,340 individuals from 24 cohorts of diverse ancestry, including 16 European cohorts (*n* = 13,266), one Middle Eastern cohort (*n* = 481), one East Asian cohort (*n* = 811), one African American cohort (*n* = 114), one US Hispanic/Latino cohort (*n* = 1,097), and four ancestry cohorts (*n* = 2,571). Spearman’s correlation test was used to identify loci affecting microbiome trait loci after accounting for age, sex, technical variables, and major genetic components. Furthermore, the identified microbiome trait loci were examined for correlations with health-related traits using gene set enrichment analysis, phenome-wide association study, and MR techniques. This major study analyzed the link between host genotypes and 211 gut bacteria (9 phyla, 16 orders, 20 orders, 35 families, and 131 genera) (23). Information regarding the GM cohort is presented in Tables S1, S2 (Supplementary Table 2).

### 2.3 GWAS data for frailty

Among the multiple tools used to identify frailty, the frailty index (FI) has been extensively validated; additionally, it has the largest research evidence base and acceptance (24), and it is a crucial predictor of negative health outcomes (25). The FI represents the proportion of health defects to all the defects considered during the aging process. Specifically, the FI is calculated by assigning each item a value between 0 and 1 according to the severity of the defect, based on the standardized scheme (when defects are dichotomous variables, frailty is graded as “no defects = 0” or “defects present = 1”; when defects are continuous variables, a more detailed grading is used, “no defects = 0,” “very good = 0.25,” “good = 0.5,” “fair = 0.75,” and “worst = 1”). Then, the FI value consists of the sum of individual accumulated defect scores divided by the total of all defect scores (FI = “individual accumulated defect scores”/"the sum of all defect scores”) (26). For example, out of a total of 50 items with a total of 50 defect scores, the FI value for an individual with an individual accumulated defect score of 10 is 10/50 = 0.2. The more defects a person has simultaneously, the weaker they are likely to be (27).

FI-associated IVs were obtained from a GWAS conducted in a large sample of 175,226 European individuals by Atkins et al. (28). Currently, this GWAS presents the most comprehensive investigation of the genetic factors underlying the FI. The sample included participants in both UK Biobank (*n* = 164,610, aged 60–70 years) and Swedish TwinGene (*n* = 10,616, aged 41–87 years). The calculation of the FI in this study was based on self-reported symptoms, disabilities, and diagnosed diseases, of which 49 and 44 items were from the UK Biobank and Swedish TwinGenestudies, respectively. The UK Biobank was conducted in England, Scotland, and Wales between 2006 and 2010, with 502,642 community volunteers aged 37–73 years being recruited. The TwinGene data were analyzed in Sweden between 2004 and 2008. All participants were of European origin and the final FI was generated using a full sample of cases with information on all frailty items. A GWAS meta-analysis of frailty (denoted by FI) was performed for all participants after adjustment for age, sex, and the first 10 principal components; moreover, chained imbalance score regression was used to estimate the level of bias in the GWAS as well as the heritability of the FI. Notably, 29 of the 49 items used by the UK Biobank have proximate items in Swedish TwinGene. The heritability of SNPs for the FI was estimated to be 11%, with 14 loci being associated with the FI (*p* < 5 × 10^−8^) (28). Detailed information on the FI sources and overlapping projects are presented in Tables S3–S6 (Supplementary Table 2).

### 2.4 Instrumental variable (IV)

The IV used in the MR analysis was an exposure-related genetic variation. Initially, we selected to use SNPs smaller than the genome-wide statistical significance threshold (5 × 10^−8^) as IVs; however, only a small amount of GM could be selected as IVs. Accordingly, to ensure that there were enough IVs for exploring the relationship between frailty and GM in order to obtain comprehensive results, we used a second threshold (1 × 10–5) to screen for SNPs (29). Subsequently, we obtained independent SNPs to calculate linkage disequilibrium with reference to the 1,000 Genomes Project European samples data, using r^2^ < 0.001 and distances >10,000 kb as criteria. Finally, we excluded SNPs with F-statistics <10 as well as ambiguous and duplicated SNPs. Details regarding the SNPs used as IVs are presented in Table S7 (Supplementary Table 2) and Table S1 (Supplementary Table 3).

### 2.5 Statistical analysis

#### 2.5.1 MR analysis

MR analyses were performed using three different MR methods: random effects inverse variance weighting (IVW), MR-Egger, and weighted median (WM). These methods were employed to address the heterogeneity and pleiotropy of the results. IVW was used as the primary outcome (30); additionally, for exposures with at least three SNP measurements, IVW under a multiplicative random effects model was used as the primary statistic. Otherwise, the IVW fixed effects method was used. In contrast, MR Egger and weighted median were used to correct the IVW results since they provide more reliable estimates in a wider range of scenarios; however, they are less efficient (wider confidence intervals) (30, 31). Notably, MR-Egger allows for a pleiotropic effect for all genetic variants but only when they are independent of the variant-exposure association (30). During the analysis, if an SNP was not found in the outcome, information regarding the SNP with which it was in strong linkage disequilibrium was used instead. Finally, reverse MR analysis was conducted in a similar manner to examine the impact of frailty on the GM composition.

#### 2.5.2 Risk factors

To test whether the risk factors that caused the results violated the principles of MR analysis, we performed search and exclusion of known SNPs associated with genetic IVs using the Phenoscanner platform,^1^ which is a large public database of genetic association studies that can be used to query the database for associations with specific variants. The relevant search results are presented in Table S8 (Supplementary Table 2) and Table S2 (Supplementary Table 3).

#### 2.5.3 Sensitivity analysis

Horizontal pleiotropy occurs when genetic variations affect outcome measures through pathways other than the exposure of interest, leading to bias of the results. To assess the reliability of the results, we used funnel plots as well as Cochran’s Q, leave-one-out (LOO), MR-Egger intercept, and MR-PRESSO tests. Specifically, a *p*-value <0.05 in Cochran’s Q-test indicated the presence of heterogeneity in our results. We used both the MR-Egger intercept and MR-PRESSO tests to assess and correct for horizontal pleiotropy; MR estimates were considered horizontal pleiotropy if the *p*-value was <0.05 (31). The MR-PRESSO assessment comprised three stages: (a) identifying the horizontal pleiotropy validity of the analyzed findings, (b) rectifying anomalies caused by horizontal pleiotropy validity by eliminating outliers, and (c) assessing consequential variations in causal estimation before and after removal of outliers.

MR-PRESSO is less biased when the horizontal pleiotropy variance is <10%; therefore, it has better accuracy than IVW and MR-Egger analyses (32). To rule out the causality of individual SNPs, we performed a LOO analysis wherein any SNPs associated with exposure were discarded and the IVW analysis was repeated. We considered causal associations to be significant when the following three conditions were met: MR estimates were significant at nominal significance (*p* < 0.05), no significant data variability was demonstrated in sensitivity analyses, and no horizontal pleiotropy was found after the MR-Egger intercept and MR-PRESSO analyses. To adjust the results for multiple comparisons, we introduced a false discovery rate (FDR) and defined statistical significance as a *q*-value <0.05 (33). Finally, we performed reverse MR analyses to assess whether frailty affected the gut flora.

MR estimates were expressed as beta values and the corresponding 95% confidence intervals, which provide an estimate of the risk of frailty due to the GM. All analyses were performed using TwoSampleMR (version 0.5.7) and MR-PRESSO (version 1.0) in R software (version 4.3.1).

## 3 Results

### 3.1 Influence of GM on frailty

On the basis of the selection criteria for IVs, we selected 2,361 SNPs as IVs of 211 bacterial genera. In the final analysis, 85 SNPs from seven bacterial genera were identified; among the 85 results, F-statistics for IVs ranged from 18.60 to 32.65, which eliminated the bias of weak IVs.

**Figure 2:**
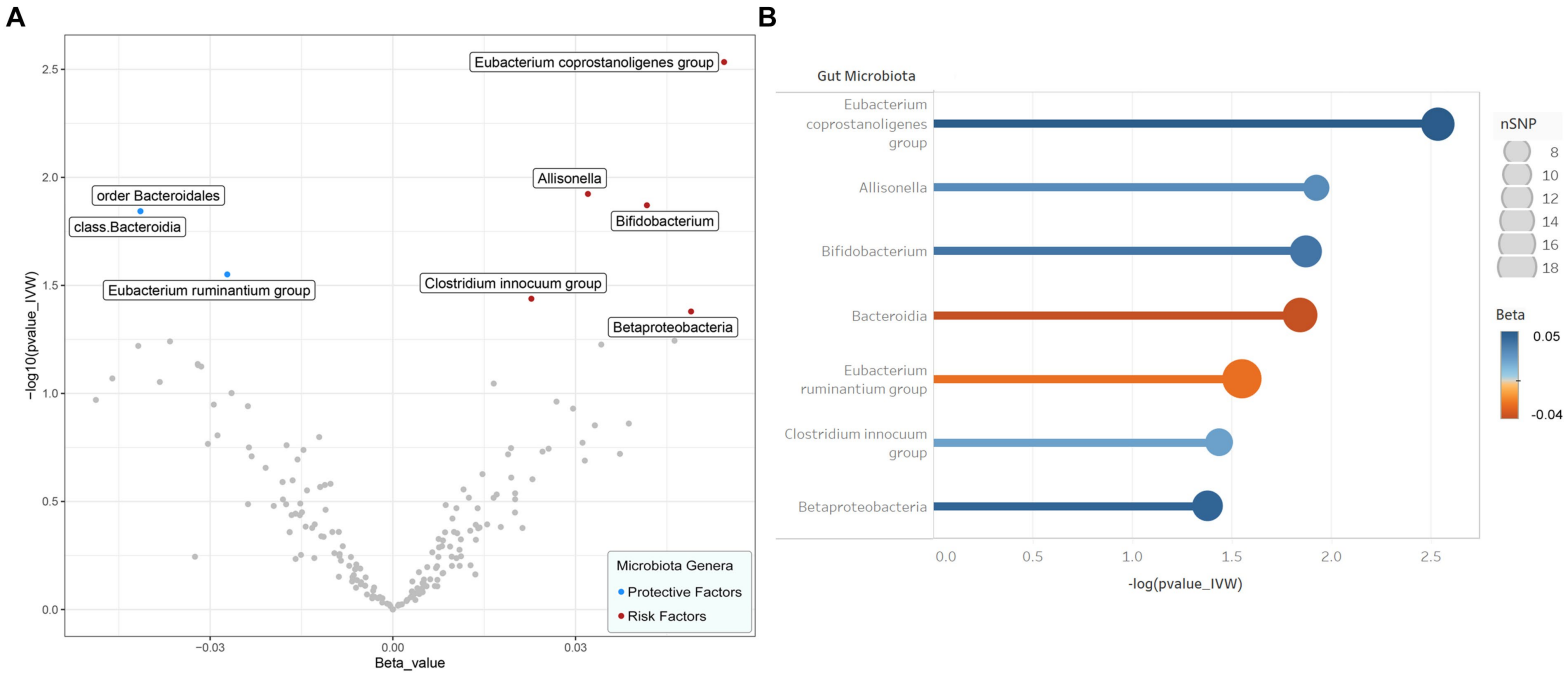
Results of preliminary IVW analyses. **(A)** The volcano plot illustrates the relationship between gut flora and risk of frailty. The X-axis represents the beta-value, the Y-axis represents the logarithmic *p*-value with a base of 10, *p* < 0.05 is considered as statistically significant. Red and green dots represent the risk and protective microbiota genera for frailty, respectively. **(B)** The lollipop plot further depicts seven statistically significant gut microbiota genera by *p*-value rank, the size of the points represents the number of SNPs, and the color of the points represents the beta-value. IVW inverse variance weighted, SNP single nucleotide polymorphism.

**Figure 3:**
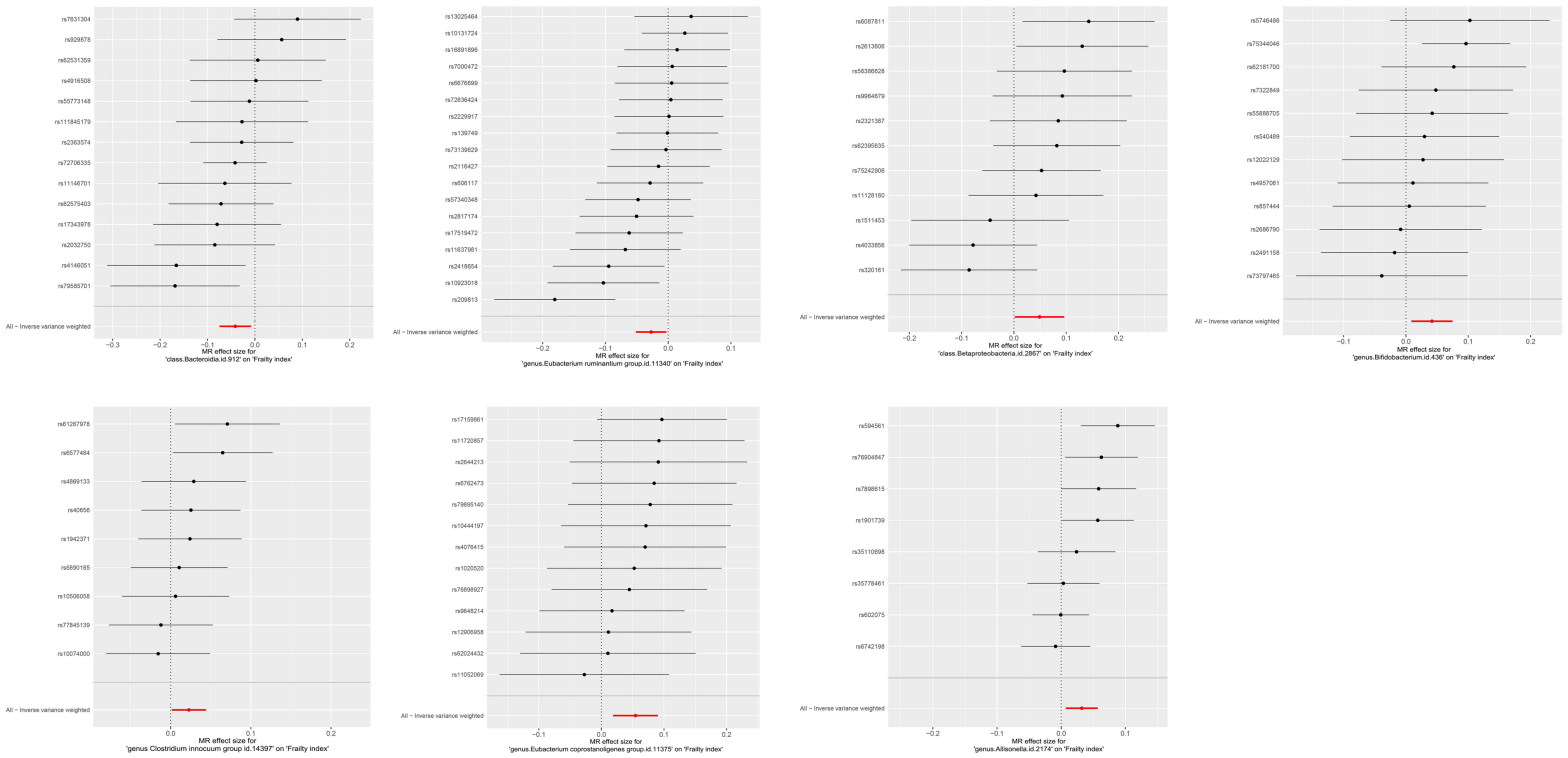
MR effect size for gut microbiota on Frailty index. Weakness progressively decreases with increasing abundance of *Bacteroidia* class and *E. ruminantium genus*, while abundance of *Betaproteobacteria class, Bifidobacterium genus, Clostridium innocuum genus, E. coprostanoligenes genus* and *Allisonella genus* increases weakness. Continuous traits are expressed as effect size β. Points indicate point estimates. Horizontal bars show 95% CI.

The IVW results were used as the main reference (*p* < 0.05). We identified a total of seven bacterial genera related to the FI, including *Bacteroidia*, *Betaproteobacteria*, *Allisonella*, *Bifidobacterium*, *Clostridium innocuum*, *Eubacterium coprostanoligenes*, *E. ruminantium*, and *Bacteroidia* (Figure 2). Among them, *Bacteroidia* (*b* = −0.041, SE = 0.017, *p* = 0.014) and *E. ruminantium* (*b* = −0.027, SE = 0.012, *p* = 0.028) were associated with low FI risk and could promote physical health in elderly individuals; however, WM and MR-Egger analysis did not support a causal relationship between GM and frailty. Contrastingly, *Betaproteobacteria* (*b* = 0.049, SE = 0.024, *p* = 0.042), *Bifidobacterium* (*b* = 0.042, SE = 0.016, *p* = 0.013), *C. innocuum* (*b* = 0.023, SE = 0.011, *p* = 0.036), *E. coprostanoligenes* (*b* = 0.054, SE = 0.018, *p* = 0.003), and *Allisonella* (*b* = 0.032, SE = 0.013, *p* = 0.012) were associated with high FI risk. Additionally, WM analysis of *Betaproteobacteria* and *E. coprostanoligenes* yielded similar results as IVW, whereas the remaining methods did not offer significant results (*p* = 0.059–0.916). In our analyses, two bacteria exhibited the same results (*Bacteroidia* and Bacteroidales); therefore, we only retained the results for *Bacteroidia*. The details of the final analysis results are presented in Figure 3 and Table S9 (Supplementary Table 2).

To ensure that the final results were robust, we performed several sensitivity analyses, including MR-Egger intercept, MR-PRESSO global, and Cochran’s Q tests. No outliers were detected using MR-PRESSO analysis (all *p*-values were > 0.05). The MR-Egger intercept test—with all *p*-values >0.05—could not reject an intercept of 0, suggesting that our MR assessment did not introduce a pleiotropic bias. Additionally, using Cochran’s Q-test, no heterogeneity was found with respect to the association between GM and FI (all *p*-values were > 0.05). In addition, LOO analyses revealed potential outliers for *Betaproteobacteria*, *C. innocuum*, and *E. ruminantium*; however, MR-PRESSO analyses revealed no significant outliers (global test *p* > 0.05). Therefore, there was insufficient evidence of horizontal pleiotropy in the analyses of the associations between these gut bacteria and frailty. These associations remained significant even after FDR correction (*p* < 0.05). Detailed information regarding the sensitivity and calibration analyses is presented in Table S10 (Supplementary Table 2). Visualizations of the results of the correlation analysis are shown in Tables S3–S5 (Supplementary Table 3).

### 3.2 Influence of frailty on GM

In the reverse analysis, seven SNPs were screened. Using the IVW results as the main reference (*p* < 0.05), the results demonstrated that *Butyrivibrio* was affected by frailty (*b* = 1.226, SE = 0.570, *p* = 0.031), with its abundance gradually increasing as the degree of frailty increased. However, MR-Egger analysis did not yield similar results (*p* = 0.166). Meanwhile, no heterogeneity or multiplicity of results was observed after performing MR-Egger intercept, Cochran’s Q, or MR-PRESSO global tests. LOO analyses revealed that no SNPs influenced the direction of the final results and that the funnel plot was symmetrical. All the above associations remained significant after FDR correction (*q* < 0.05). The results of this analysis are presented in Tables S3–S5 (Supplementary Table 2). Visualizations of the results of the reverse analysis are shown in Supplementary Figure S4.

## 4 Discussion

With the gradual increase in the aging population in recent years, numerous health issues—among which frailty is particularly pronounced—have emerged, adding to the societal burden. Understanding the essential pathophysiology contributing to frailty is critical to revealing certain mechanisms that can be targeted. In this study, we used GM and FI data from a two-sample MR analysis to assess the association between the GM and frailty. The GM and FI data used in the analyses were derived from summary statistics of the largest GWAS meta-analysis conducted by the MiBioGen Consortium and a study published by Atkins et al. (28). Our findings indicated that *Bacteroidia* and *E. ruminantium* had a beneficial impact on the health of elderly individuals by lowering the FI. Conversely, the presence of *Betaproteobacteria*, *Bifidobacterium*, *C. innocuum*, *E. coprostanoligenes*, and *Allisonella* was correlated with an increased FI, which may be detrimental to health. Further, our findings suggested that a high FI may increase the abundance of *Butyrivibrio*. Overall, our study provides new insights into the association between specific bacterial characteristics and frailty onset. Accordingly, it may be possible to prevent and treat frailty by targeting specific bacterial communities.

Several studies have demonstrated a link of dysbiosis in the gut microbial ecology with changes in muscle mass and function that lead to reduced muscle function and strength. Decreased activity levels are a sign of frailty; moreover, the related loss of muscle mass and strength can directly lead to decreased physical activity levels (1). Animal studies have shown that ecological dysregulation of GM populations can increase intestinal permeability, which promotes the entry of endotoxins and other microbial products (e.g., lipopolysaccharides) into circulation (34), inducing muscle inflammation and insulin resistance, and therefore affect skeletal muscle metabolism and contractile function (14, 35). Systemic inflammation is implicated in the pathophysiology of sarcopenia (36). In addition, changes in microbial composition lead to changes in microbial metabolites, which are equally important in the regulation of host physiological metabolic processes. SCFAs are carboxylic acids produced by intestinal bacteria in the cecum and colon through the fermentation of dietary fiber. They mainly include acetate (C2), propionate (C3), and butyrate (11); further, they are positively correlated with the prevalence of *Bacteroidia* and *E. ruminantium* and negatively correlated with the prevalence of *Bifidobacterium* (37, 38). SCFAs can promote glucose uptake and oxidative metabolism in the mitochondria of skeletal muscle cells, which prevents muscle loss and increased intramuscular fat deposition, as well as maintains skeletal muscle function, to a certain extent (39). Regarding skeletal muscle metabolism, *Synechococcus* spp. and *E. ruminantis* had an inhibitory effect on frailty, whereas *Bifidobacterium* spp. had a facilitatory effect on frailty, which is consistent with our results. Although these findings infer the role of the gut-muscle axis in frailty, further studies are warranted to elucidate the precise mechanisms.

In this study, our findings demonstrated the positive impact of *Bacteroidia* in reducing frailty. *Mycobacterium anthropophilum* is the most prevalent and abundant member of the mammalian GM; additionally, it is an important bacterium for maintaining body homeostasis. Studies have indicated that the abundance of *Bacteroidia* tend to decrease in elderly individuals with frailty, reducing from 11 to 4.5%; moreover, it decreases by more than tenfold in hospitalized elderly patients (40, 41). They compete with pathogens for host-derived amino acids (proline and hydroxyproline) and monosaccharides (ribose, fucose, arabinose, rhamnose, and fructose) as well as produce SCFAs, and therefore inhibit pathogenesis (42). In addition, *Bacteroidetes mimosus* modulates endothelial cell function and reduces inflammation; further, it promotes CD4 T cell development through the expression of polysaccharide A (42, 43), which is crucially involved in colonic motility, immune control, and suppression of intestinal inflammation (44). Regarding nutrient metabolism, a study on maternal body weight showed that the abundance of *B. anomalosa* was positively correlated with plasma biomarkers of lipid metabolism, which contributed to the suppression of metabolic dysfunction (13). Our findings are consistent with these previous reports, indicating an association between *Bacteroidia* and frailty. Notably, our findings did not reveal an established marker of frailty (i.e., a decrease in Prevotella, which is a member of the *Bacteroidia* class) (40, 45).

Additionally, our results suggest that *Bifidobacterium* is detrimental to health, which is consistent with the results reported by Almeida et al. (16). Bifidobacteria are important commensal bacteria that regulate gut and immune system functions. In most previous studies, *Bifidobacterium* was found to modulate host defense responses, prevent infectious diseases, and inhibit Shiga toxin-producing *Escherichia coli* through acetic acid production in the body. Therefore, they have been considered beneficial for health and longevity (46); however, they can act as opportunistic pathogens, causing infections and excessive immune stimulation in immunocompromised populations (47) [such as increased levels of *Bifidobacterium* in the guts of patients with Parkinson’s disease (48)]. Additionally, *Bifidobacterium* as a probiotic additive can cause septicemia in young children (49).

*Clostridium innocuum* is mainly associated with metabolism and may be crucially involved in sepsis; further, its abundance is increased in patients with acute gastrointestinal tract injury (50). Interestingly, several other animal studies have demonstrated beneficial effects of *C. innocuum*. In mice with hyperlipidemia induced by a high-fat diet, increasing the abundance of *C. innocuum* effectively reduced the weight of adipose tissue and ameliorate lipid metabolism disorders (51, 52). These findings highlight the complex role of *C. innocuum*.

*Betaproteobacteria* and *E. coprostanoligenes* may have beneficial effects on the FI. Despite the lack of direct observational evidence of an association of these two bacteria with frailty, previous studies have suggested that these bacteria are inextricably linked to the development of a range of conditions, including decompensated cirrhosis, malignant cancers, unexplained chronic kidney disease, and arthritis (53–56).

Hosts and their gut microbial communities form complex ecosystems. In previous animal studies, age and frailty affected the composition and function of gut microbial communities (57). Similarly, we found that frailty could alter the composition of gut microbial communities. Frailty has been demonstrated to dramatically affect the composition and structure of the gut microbial community by modulating host metabolism (e.g., Lactobacillus intake and defecation frequency) (40, 58). Our inverse MR analyses confirmed the causal effect of frailty-related traits on GM clusters, indicating a reciprocal causal relationship between the GM clusters and frailty.

Our genetic analyses of the GM and frailty were based on extensive GWAS association studies. This technique can effectively reduce confounding impacts, such as environmental and lifestyle factors, and therefore enhance the reliability of the findings. Moreover, we ensured the robustness of our results by detecting and excluding horizontal pleiotropy using MR-PRESSO and MR-Egger regression intercept term tests. Our results suggest that changes in the GM may affect human health; however, the association between the two remains unclear. The links between the GM and frailty in existing studies remain inconclusive, which could be attributed to among-study differences in the sample sizes, geographic backgrounds, dietary habits, ancestry, and age of the participants.

This study has several limitations. First, we used data available in the GWAS database to determine IVs; however, the number of genome cluster SNPs was limited, and some bacteria were not included. Thus, we could not completely rule out potential causality. Larger bacterial GWAS studies are needed to achieve sufficient statistical power. Second, the bacterial GWAS data included multiple ancestries, whereas the GWAS data on frailty were only obtained populations of European ancestry, which limits the generalizability of the findings to fragile populations in other pedigrees. In the future, the data on frailty from populations of other ancestries are required for more in-depth and comprehensive analyses. Third, the heritability explained by the eight species of gut bacteria we studied was not high enough, resulting in the lack of good genetic correlations. However, the results of our Mendelian randomization analyses were robust in multiplicity and sensitivity analyses; therefore, we believe that this study’s results are still suggestive of a potential causal relationship between GM and frailty (59). Finally, the GM is large and complex in function, and this study did not identify a specific mechanism through which the GM interacts with frailty. Therefore, additional samples are required to further investigate the relationship and mechanisms of the role of flora in frailty.

## 5 Conclusion

This study indicated a robust link between the GM and frailty. Alterations in microbiological composition may trigger frailty-related health issues, primarily in senior communities. Nevertheless, frailty is a multifaceted problem influenced by numerous factors; therefore, additional RCTs are warranted to clarify the protective function of GM against frailty and the underlying mechanisms, as well as to identify plausible interventions that enhance the health status of older adults and improve their quality of life.

## Data availability statement

The original contributions presented in the study are included in the article/Supplementary material, further inquiries can be directed to the corresponding author.

## Ethics statement

Ethical approval was not required for the study involving humans in accordance with the local legislation and institutional requirements. Written informed consent to participate in this study was not required from the participants or the participants’ legal guardians/next of kin in accordance with the national legislation and the institutional requirements.

## Author contributions

FB: Conceptualization, Writing – original draft. HT: Conceptualization, Writing – review & editing. JS: Methodology, Writing – review & editing. ZL: Data curation, Methodology, Writing – review & editing. YXu: Methodology, Writing – review & editing. RP: Formal analysis, Writing – review & editing. YXi: Formal analysis, Writing – review & editing. SZ: Formal analysis, Writing – review & editing. YZ: Supervision, Writing – review & editing. WZ: Funding acquisition, Supervision, Writing – review & editing.

## Footnotes

^1^www.phenoscanner.medschl.cam.ac.uk
