# Supplementary material for: Exploring the causal relationship between gut microbiota and frailty: a two-sample mendelian randomization analysis
Source: Front Med (Lausanne). 2024 May 3;11:1354037. doi: 10.3389/fmed.2024.1354037 (PMC11099276; doi:10.3389/fmed.2024.1354037)
Supplement: Supplementary file 1 [file Table_1.docx]

**Exploring the causal relationship between gut microbiota and frailty: a two-sample Mendelian randomization analysis**

**Fuduo Bo ^1†^, Hong Teng ^1†^, Jianwei Shi ^2^, Zhengxiang Luo ^1^, Yang Xu ^1^, Ruihan Pan ^3^, Yan Xia ^1^, Shuaishuai Zhu ^1^, Yansong Zhang ^1*^ and Wenbin Zhang ^1*^**

Figure S1. Scatter plots for Influence of gut microbes on frailty.

Figure S2. Funnel plots for Influence of gut microbes on frailty.

Figure S3. Leave-one-out analysis for Influence of gut microbes on frailty.

Figure S4. Forest Plots, Scatter plots, Funnel plots and Leave-one-out analysis for Influence of frailty on gut microbes.
